# Severe Eosinophilia in Myelodysplastic Syndrome With a Defined and Rare Cytogenetic Abnormality

**DOI:** 10.3389/fimmu.2018.03031

**Published:** 2019-01-09

**Authors:** Shinya Rai, J. Luis Espinoza, Yasuyoshi Morita, Hirokazu Tanaka, Itaru Matsumura

**Affiliations:** Department of Hematology and Rheumatology, Faculty of Medicine, Kindai University Hospital, Osaka-Sayama, Japan

**Keywords:** eosinophilia, cytogenetic (CG) analyses, eosinophilic pneumonia, myelofibrosis, membranoproliferative glomerulonephritis (MPGN), myelodisdplastic/myeloproliferative disorders

## Abstract

Myelodysplastic syndromes (MDS) are a heterogeneous group clonal disorders of hematopoietic stem cells (HSC) characterized by ineffective hematopoiesis that lead to variable grades of impaired blood cell production. Chromosomal aberrations are often detected in MDS patients and thus cytogenetic analysis is useful for the diagnosis of these disorders. Common recurring chromosomal defects, such as the −5/5q- and −7/7q- are relatively well characterized cytogenetic abnormalities in MDS, however, the biological significance of uncommon cytogenetic alterations is unknown. We report here, two cases of peripheral blood and bone marrow hypereosinophilia in patients with MDS harboring the unbalanced translocation der(1;7)(q10;p10), a poorly characterized cytogenetic abnormality that is found in certain myeloid malignancies, including MDS. The patients reported here presented hypereosinophilia that was refractory to steroids and cytotoxic therapy, leading to severe target tissue damage that ultimately resulted in fatal end-organ failure. Potential roles of the der(1;7)(q10;p10) aberrations in the pathogenesis of aggressive eosinophilia and disease prognosis are discussed here.

## Introduction

Eosinophilia, defined as a peripheral blood eosinophil count exceeding 500 μl, can be associated with a large number of disorders and is causally classified into two general categories, (1) primary eosinophilia that results from disorders that are intrinsic to the eosinophil lineage and (2) secondary eosinophilia, which is caused by factors outside the eosinophil lineage (1). In most cases secondary eosinophilia represents a reaction to the overproduction of the eosinophilopoietic cytokines such as interleukin 3 (IL-3), interleukin 15 (IL-5), and granulocyte colony stimulating factor (GM-CSF), such as the eosinophilia observed in response to allergens, drugs or parasitic infections. Secondary eosinophilia may also develop in the context of autoimmune disorders or certain malignancies such as Hodgkin's lymphomas (2, 3).

Myeloid neoplasms, including myeloproliferative neoplasms (MPN), acute myeloid leukemia (AML) and MDS are well-known causes of primary eosinophilia, in which defined clonal disorders of HSC lead to the overproduction of eosinophils (4). Hence, myeloid malignancies carrying dysregulated fusion tyrosine kinase genes (TK), including platelet-derived growth factor (PDGF-AB), fibroblast growth factor receptor 1 (FGFR1), and the PCM1-JAK2 fusion product has been recently recognized by the World Health Organization (WHO) category of myeloid neoplasms with eosinophilia and rearrangement of PDGFRA, PDGFRB, or FGFR1, or with PCM1-JAK2. These distinctive group of myeloid neoplasms present with eosinophilia, ranging from a mild increase in eosinophil count to a marked eosinophilia that can be directly responsible for target organ damage (1). Hypereosinophilia (HE) is defined as a persistent eosinophil count in the peripheral blood that exceeds 1,500 μl and may be associated with life-threatening organ damage as a result of tissue infiltration by eosinophils and the release of their granular contents (5, 6).

MDS are clonal disorders of HSCs characterized by the ineffective blood cells production leading to variable grades of cytopenias in the circulating blood (7). Overall, the incidence of MDS ranges from 3 to 5 per 100,000 individuals, however, in individuals over 70 years old, the incidence increases to about 50 per 100,000 inhabitants (8).

Typically, the bone marrow (BM) of patients with MDS shows increased cellularity with variable grade of dysplasia of bone marrow cells, ranging from erythroid hyperplasia to defective maturation in the myeloid series an increase in the number of blasts, thus patients with MDS have an exceptional increased risk for progression to AML and although most MDS cases arise *de novo*, some cases are caused by mutagenic insults, such as the prolonged exposure of the BM to cytotoxic chemotherapy (9–11).

Chromosomal abnormalities are detected in approximately 30–70% of MDS patients and the presence of cytogenetic abnormalities correlates with disease prognosis, consequently, disease karyotype constitutes an important component of the International Prognostic Scoring System (IPSS) and the revised IPSS-R (IPSS-R), which are the most broadly utilized scoring tools for assessing prognosis in patients with MDS (11, 12). Notably, the prognostic relevance of cytogenetic abnormalities in MDS is largely limited to the most frequent ones, including del(5q),−7/del(7q),−8,-18/del (18q), del (20q),-5,-Y,-17/del (17p) (12, 13) and hence the biological relevance of less common chromosomal alterations in MDS is poorly understood. This is particularly important considering that over 600 different cytogenetic categories had been identified in MDS patients (14).

The unbalanced translocation, der(1;7)(q10;p10), is a rare cytogenetic abnormality that is detectable in some myeloid malignancies, including MDS (1–3%), AML (1–2%), and MPN (1%) (15, 16). MDS patients harboring this translocation appear to have specific molecular and clinical features, however, given its scarceness compared with better characterized cytogenetic groups found in AML/MDS, several aspects of this entity have not been described (15, 17).

Here, we report two MDS cases with (1;7)(q10;p10) translocation in previously healthy men who presented with severe hypereosinophilia and target organ damage leading to fatal complications. In both cases, a large number of eosinophils were detectable in the peripheral blood and BM and eosinophilia was refractory to the treatment with corticosteroids.

## Case 1

A previously healthy 67-year old male presented to another hospital complaining of dry cough, wheezing and mild dyspnea. Physical examination was unremarkable, except for the signs of bronchoconstriction. The laboratory tests revealed a marked increase in the number of eosinophils in the peripheral blood and thus a diagnosis of eosinophilic asthma was made. He was given inhaled bronchodilators and corticosteroids which induced a moderate improvement of symptoms. Four months later his symptoms worsened and was then diagnosed as Chronic Eosinophilic Pneumonia and oral methylprednisolone was added, which induced a minor improvement of symptoms without affecting eosinophilia. In addition, dry cough and respiratory discomfort reoccurred along with tapering the methylprednisolone to 10 mg/day. He was referred to our hospital in July 2016 for further evaluation. He had no smoking history and his medical history was unremarkable. On examination, vital signs were stable except for requiring 1L of nasal cannula oxygen. The SaO2 was 96% on 1L oxygen. He had decreased breath sounds in the lower right lung field with fine crackles. He had no raised JVP, murmurs, gallop or peripheral edema. Chest x-ray revealed right ground glass opacities (GGOs). A high-resolution CT scan revealed GGOs surrounded by consolidation in the right lower lung field.

Main laboratory findings were as follows: WBC 7,770/μl, with eosinophils 52.3%; red blood cells (RBC) 366 × 10^4^/μl; hemoglobin (Hb) 8.6 g/dl; Platelets (Plt) 25.5 × 10^4^/μl; C-reactive protein 2.66 mg/dl (normal <0.3 mg/dl); lactate dehydrogenase (LDH) 243 IU/L (normal range <225 IU/L); IgE 254 IU/ml (normal <232 IU/ml); Soluble IL-2 receptor (sIL-2R) 495 U/ml (normal 150–505 U/ml); serum thymus and activation-regulated chemokine (TARC) 119 pg/mL (Table 1).

**Table 1:** Laboratory test at the diagnosis in case 1.

**(Peripheral blood)**		**(Biochemistry)**			
WBC	7,700/μL	CRP	2.66 mg/dL	ANA	Negative
Stab	5.0%	Ca	8.8 mg/dL	Jo-1-Ab	Negative
Seg	33.5%	UA	4.8 mg/dL	RF	<7.0 U/mL
Eo	53.5%	BUN	12.0 mg/dL	PR-ANCA	<1.0 U/mL
Baso	4.0%	Crea	0.90 mg/dL	MPO-ANCA	<1.0 EU
Mono	2.5%	TP	6.6 g/dL	TARC	119 pg/mL
Lymph	1.5%	Alb	3.5 g/dL		
RBC	366 × 10^4^/μL	AST	15 U/L		
HGB	8.6 g/dL	ALT	11 U/L	(Arterial blood gas analysis)	
PLT	25.5 × 10^4^/μL	LDH	243 U/L	pH	7.549
(Infectious marker)	ALP	176 U/L	pCO2	27.1 mmHg	
HBs-Ag	(–)	T-Cho	156 mg/dL	pO2	68.2 mmHg
HBs-Ab	(–)	TG	86 mg/dL	HCO3	23.1 mmol/L
HCV-Ab	(–)	sIL-2R	495 U/mL		
HIV-Ab	(–)	IgE	254 IU/mL		

*ANA, anti-nuclear antibody; RF, rheumatoid factor; PR3-ANCA, proteinase3-antineutrophil cytoplasmic antibody; MPO-ANCA, myeroperoxidase-antineutrophil cytoplasmic antibody; TARC, Thymus and activation-regulated chemokine*.

**Figure 1:**
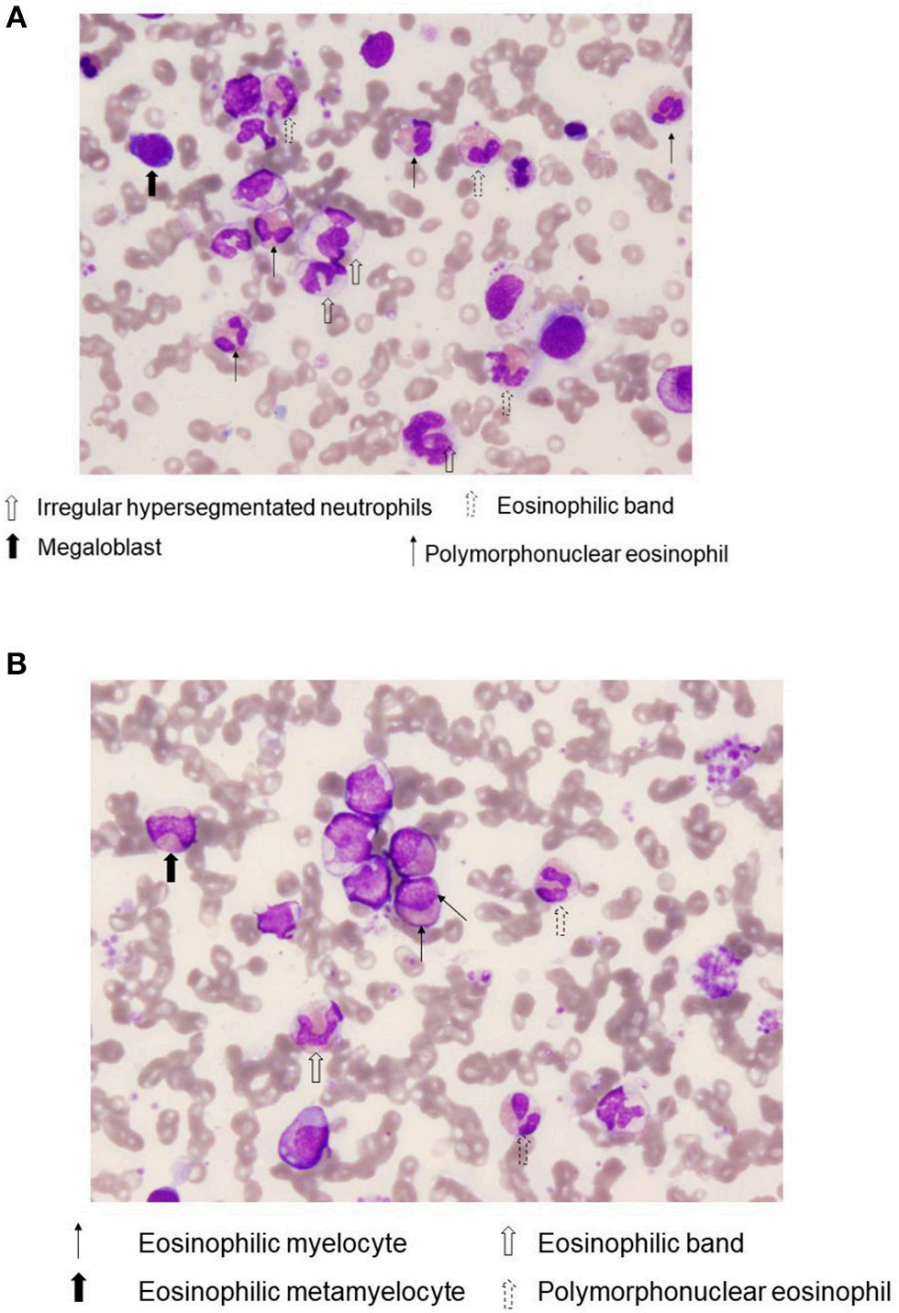
Bone marrow eosinophilia. A representative Wright-Giemsa stain, (×400) of bone marrow aspirate smears from case 1. Notice the presence of various immature eosinophils, including eosinophilic band and Polymorphonuclear eosinophils **(A)**, as well as eosinophilic myelocyte and eosinophilic metamyelocyte **(B)**.

**Figure 2:**
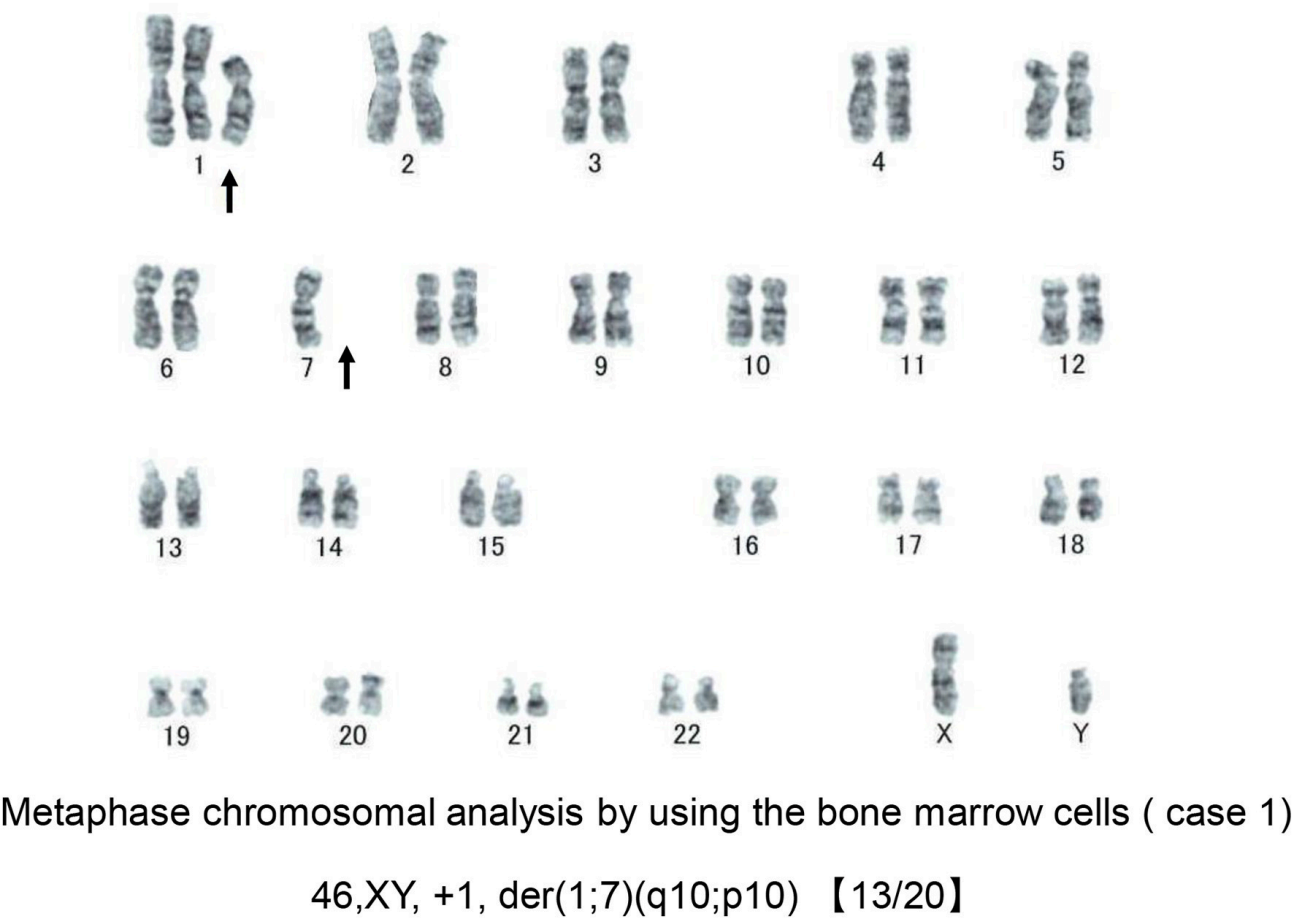
Chromosomal analysis of case 1. A representative metaphase chromosomal analysis of the bone marrow cells derived from case 1. Arrows indicate the allelic imbalance of trisomy 1q and monosomy 7q.

Bone marrow (BM) aspirate demonstrated infiltration of eosinophils (26.2% of the total BM cells) without dysplasia. The percentage of blast cells in the BM was 2.4% with trilineage dysplasia seen in megakaryocytes, as well as in myeloid and erythroid lineages (Figures 1A,B). Chromosomal analysis of BM cells showed 46, XY, +1, der(1;7) (q10;p10) in 13 of 20 metaphases (Figure 2). He was negative for PDGFRA, PDGFRB rearrangement or FGFR1, or with JAK2 mutations. Accordingly, a diagnosis of MDS (refractory cytopenias with multilineage dysplasia type) was made, and consequently was categorized as intermediate risk, according to IPSS-R and intermediate-1 risk according to the IPSS scoring.

**Figure 3:**
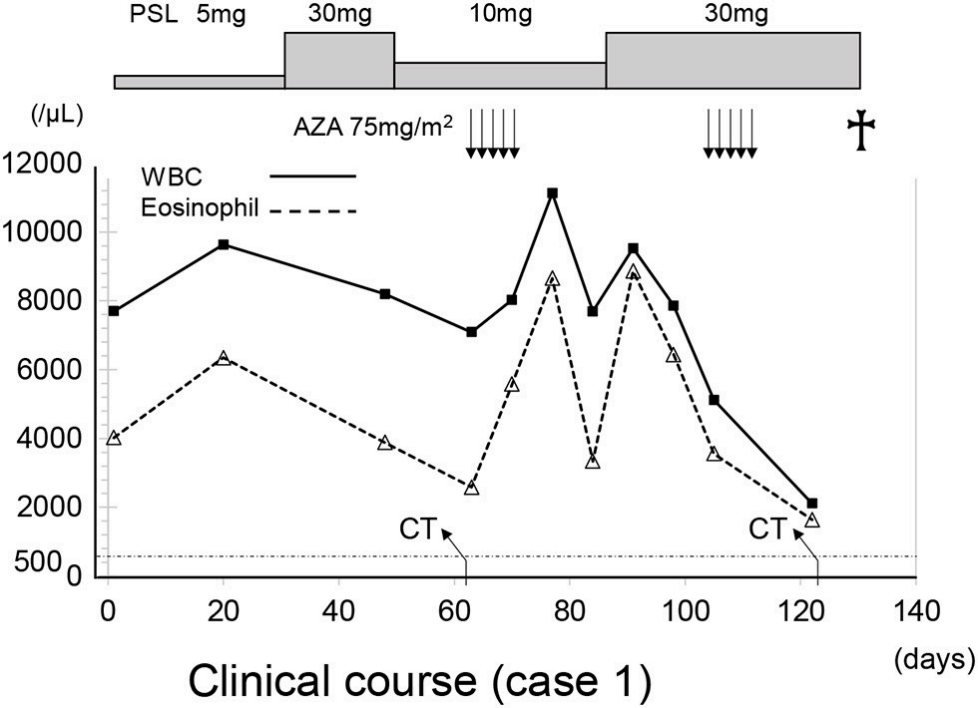
The clinical course of case 1. The X axis indicate the time (days). AZA, azacitidine; WBC, white blood cells; PLS, prednisolone.

**Figure 4:**
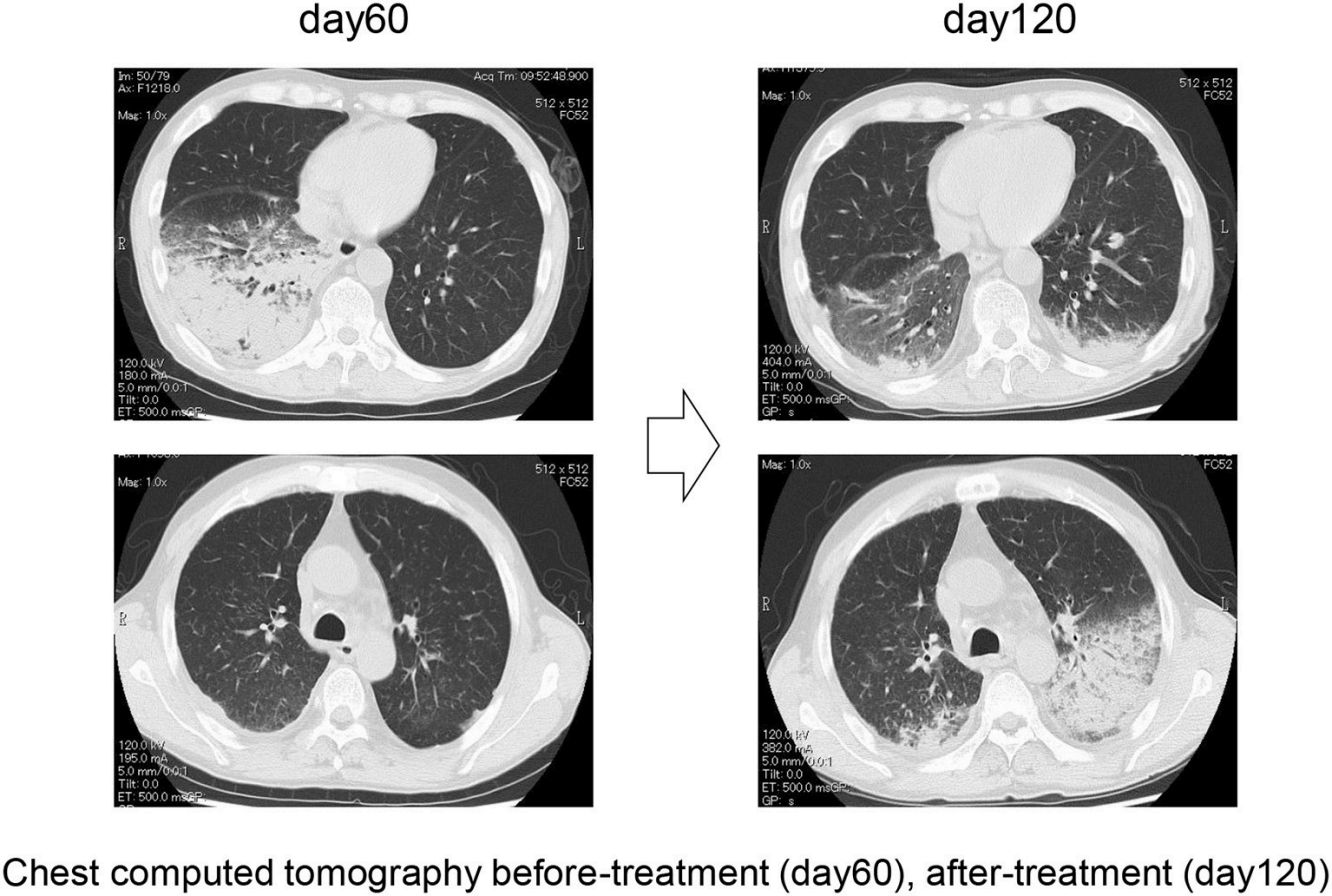
Representative chest computer tomography of case 1, showing consolidation in the right lower lung field at day 60 and consolidation in the left lower lung field at day 120.

The patient was treated with 75 mg/m^2^ azacitidine (AZA) once daily for five consecutive days on a 28-day cycle based (Figure 3). After the first cycle of AZA, the number of eosinophils further increased (WBC 6,570/μl with eosinophils 60.2%), which coincided with the tapering of 10 mg/day. After completing the second cycle of AZA treatment and increasing prednisolone to 30 mg/day, we noticed a substantial decrease in the right lung infiltrate. However, before starting the third cycle of AZA, dyspnea, cough and wheezing significantly worsened and a new infiltrate was detected in the left lower lung field (Figure 4). The infiltrate was refractory to various antimicrobial regimens combined with methylprednisolone and the patient's condition deteriorated, leading to respiratory failure and death.

## Case 2

A 23-year old male presented to our hospital in April 2005 with maculopapular rash involving >50% of his body and intermittent fever of several weeks of evolution. He had no significant past medical history and denied symptoms of fatigue, body weight loss, or night sweats. Physical examination was unremarkable, except for the presence of a maculopapular rash covering nearly 50% of the skin surface.

**Figure 5:**
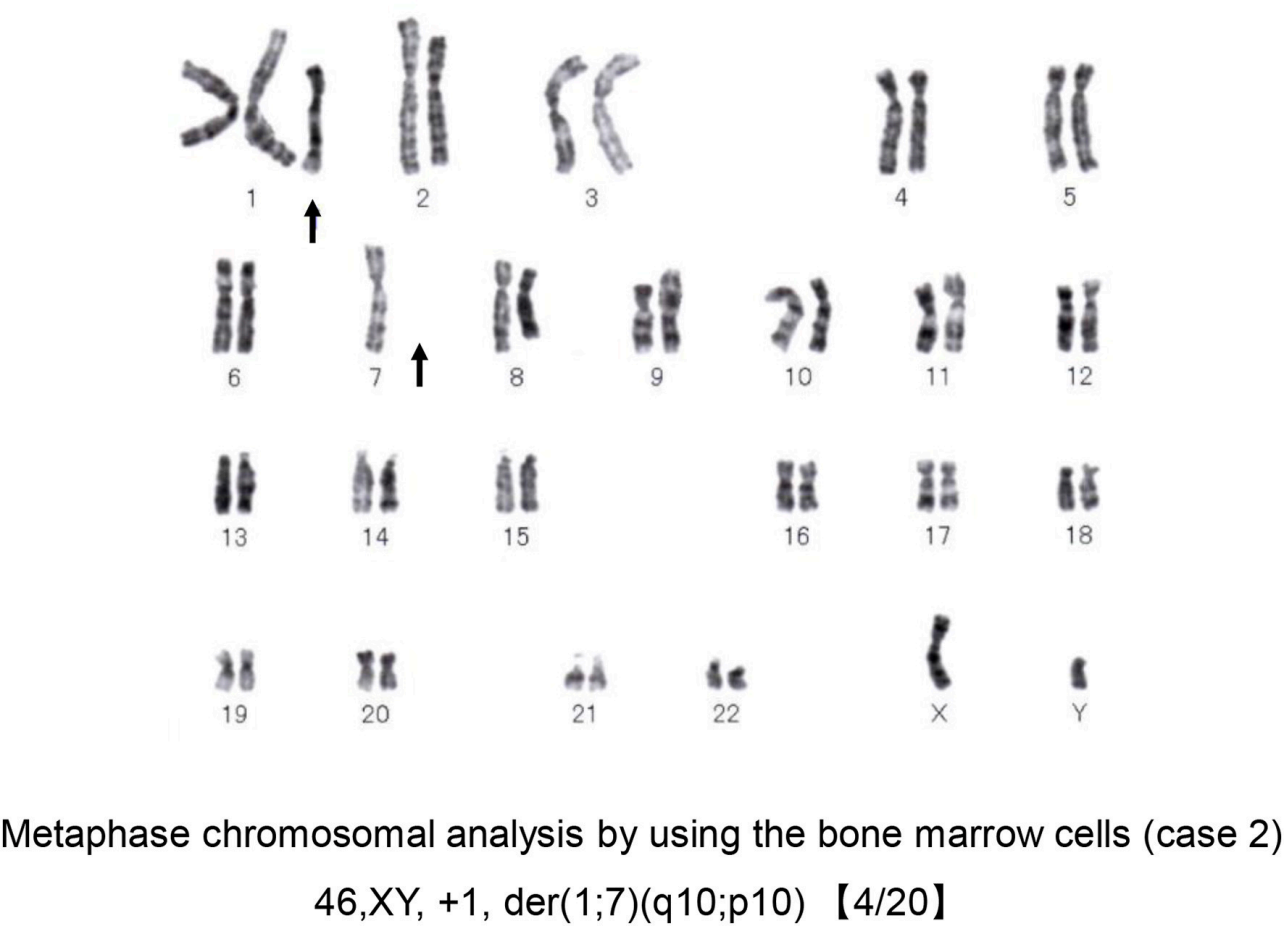
Chromosomal analysis of case 2. A representative metaphase chromosomal analysis of the bone marrow cells derived from case 2. Arrows indicate the allelic imbalance of trisomy 1q and monosomy 7q.

Laboratory findings were as follows: WBC 24,300/μl with eosinophils 39.0%; RBC 263 × 10^4^/μl; Hb 10.0 g/dl; Plt 12.5 × 10^4^/μl; C-reactive protein 1.66 mg/dl (normal <0.3 mg/dl); LDH 363 IU/L (normal range <225 IU/L); creatinine 0.95 mg/dl and estimated glomerular filtration rate (eGFR) of 65.7 ml/min/1.73 m^2^ (according to the modification of the CKD Epidemiology Collaboration Equation for Japanese); IgE 1,156 IU/ml (normal <232 IU/mL). A BM aspirate demonstrated significant infiltration of eosinophils (23% of total BM cells) without dysplasia and a 0.3% of blast cells with dysplasia in the erythroid lineages. Chromosomal analysis of BM cells showed 46, XY, +1, der(1;7) (q10;p10) in 4 of 20 metaphases (Figure 5). He was negative for PDGFRA, PDGFRB rearrangement or FGFR1, or with JAK2 mutations.

A diagnosis of MDS (Refractory anemia type) with hypereosinophilic syndrome (HES**)** was made and was subsequently categorized as low risk according to IPSS-R and intermediate-1 according to the IPSS scoring.

**Figure 6:**
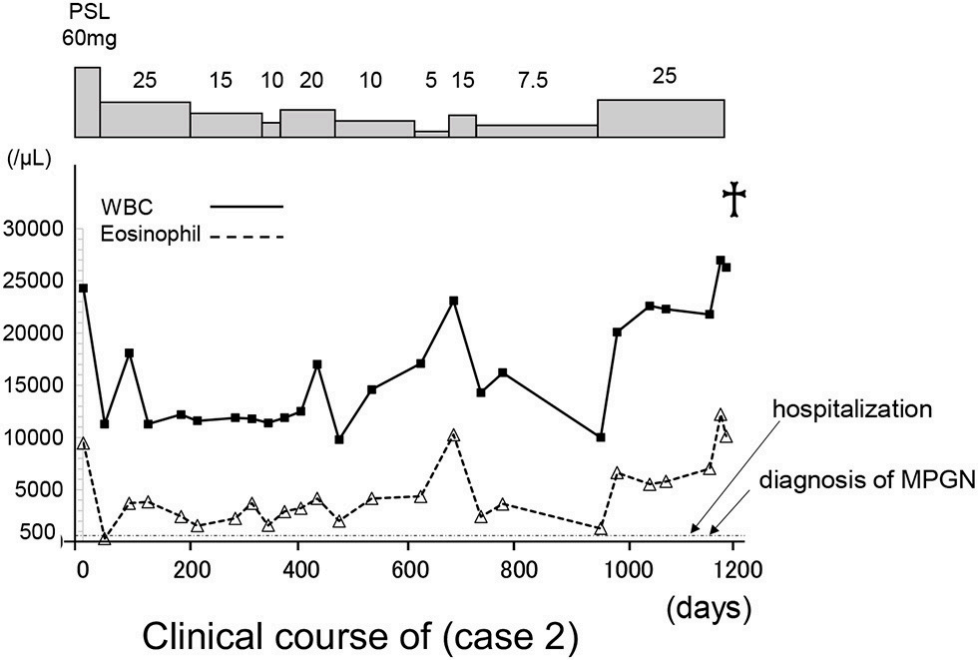
The clinical course of case 2. The X axis indicate the time (days). WBC, white blood cells; PLS, prednisolone.

**Figure 7:**
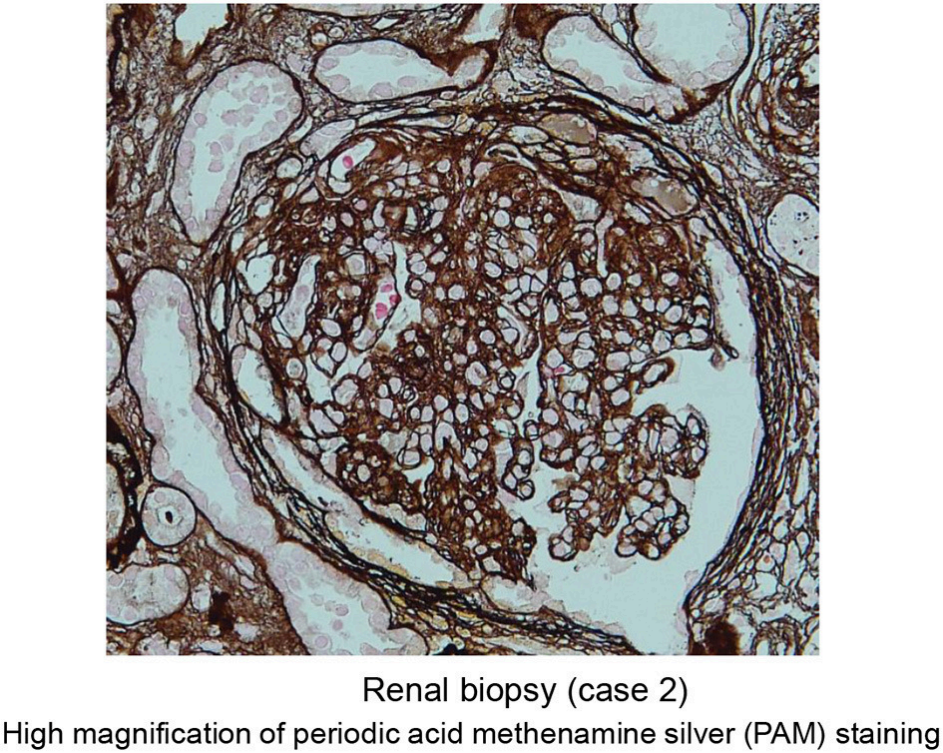
Representative images of periodic acid methenamine silver staining (high magnification) of renal specimen from patient 2 showing duplication of the capillary wall consistent with membranoproliferative glomerulonephritis.

He was initially treated with methylprednisolone (1.0 mg/kg/day) in an attempt to control HES. Within approximately 1 week, significant improvement in the clinical condition was observed, and eosinophil count returned to normal values. However, the maculopapular rash and eosinophilia reoccurred along with tapering the methylprednisolone to 25 mg/day along with a rapid and progressive increase in the serum levels of creatinine (7.61 mg/dl) (Table 2 and Figure 6). A renal biopsy was performed for histopathological diagnosis, which showed globally sclerotic glomerulus in four out of 14 glomeruli analyzed by light microscopy. The remaining 10 glomeruli appeared enlarged and the marked diffuse thickening of glomerular basement membranes (GBM) and mesangial cell proliferation were also noted. Mesangial interposition neither endocapillary hypercellularity nor crescent formation in these glomeruli were noted periodic acid methenamine silver staining revealed duplication of the capillary wall (Figure 7). A Congo red staining for amyloid protein was negative. A diagnosis of membranoproliferative glomerulonephritis (MPGN) was made, however the patient's condition deteriorated and died from septicemia.

**Table 2:** Laboratory test at the diagnosis of MPGN in case 2.

**(Peripheral blood)**		**(Biochemistry)**			
WBC	22,300/μL	CRP	15.18 mg/dL	ANA	Negative
Stab	1.3%	Ca	7.6 mg/dL	RF	<7.0 U/mL
Seg	43.3%	UA	4.8 mg/dL	C3	73 mg/dL
Eo	26.0%	BUN	53.0 mg/dL	C4	25 mg/dL
Baso	0.0%	Crea	7.61 mg/dL	CH50	40.1 U/mL
Mono	9.3%	TP	5.3 g/dL	PR-ANCA	<1.0 U/mL
Lymph	4.3%	Alb	2.4 g/dL	MPO-ANCA	<1.0 EU
RBC	290 × 10^4^/μL	AST	22 U/L	Anti-GBM	<2.0 U/mL
HGB	9.4 g/dL	ALP	37 U/L	(Urinalysis)	
PLT	17.8 × 10^4^/μL	LDH	536 U/L	pH	5.5
(Infectious marker)	ALP	700 U/L	Protein	3+	
HBs-Ag	(–)	T-Cho	204 mg/dL	Sugar	±
HBs-Ab	(–)	HDL-Cho	29 mg/dL	RBC	30–49/HPF
HCV-Ab	(–)	TG	182 mg/dL	WBC	1–4/HPF
HIV-Ab	(–)	sIL-2R	4,273 U/mL	(Urine chemistry)	
		IgG	502 mg/dL	NAG	10.0 U/L
		IgA	130 mg/dL	β-2MG	54,985 μg/day
		IgM	79 mg/dL	Protein	7.9 g/day
		IgE	6,760 IU/mL		

*C3, complement 3; C4, complement 4; CH50, complement hemolytic activity; Anti-GBM, anti-glomerular basement membrane*.

## Discussion

Cytogenetic aberrations are frequently detectable in patients with MDS. Some of those molecular changes have been linked to disease pathogenesis and their presence is utilized for assessing disease prognosis (13). Here we report two male patients who presented with *de novo* MDS, harboring the unbalanced translocation der(1;7)(q10;p10) associated with aggressive hypereosinophilic syndromes. In both cases, eosinophilia was not responsive to corticosteroids treatment and ultimately lead to severe target tissue damage and fatal end-organ failure.

In contrast to other hematological malignancies, in which specific chromosomal arrangements are distinctive molecular features of the disease, MDS are frequently associated with a variable number of cytogenetic abnormalities, which appear to determine the heterogeneous clinical phenotype of these disorders (18, 19).

A recurrent molecular characteristic of MDS is the loss of genetic material, via deletions and monosomies, while the gain of genetic material is uncommon. Consequently, such a loss of genetic material is consistent with the assumption that the deletion or inactivation of tumor suppressor genes, rather than the activation of oncogenes, constitutes the main molecular mechanism implicated in the development of MDS (12, 20).

The unbalanced translocation, der(1;7)(q10;p10), is a nonrandom chromosomal abnormality that occurs through a mitotic recombination between chromosome 1 and chromosome 7 that generates two copies of chromosome 1 and a single copy of the intact chromosome 7 leading to an allelic imbalance of trisomy 1q and monosomy 7q. (15–17).

There is some controversy regarding the prognosis of der(1;7)(q10;p10). Early reports involving small numbers of patients with der(1;7)(q10;p10) suggested this entity correlates with unfavorable prognosis and increased risk of progression to AML (21, 22), however, in subsequent studies that included relatively larger number of cases, the presence of this translocation in MDS indeed correlated with a better clinical outcome, with patients showing milder anemia and lower blast counts at diagnosis and a tendency to have less trilineage dysplasia and a slower progression to AML (15, 17). Similarly, in a more recent study, newly diagnosed MDS patients with der(1;7)(q10;p10) were less likely to have excess blasts or multilineage dysplasia and overall showed higher hemoglobin levels compared to patients with monosomy 7 or those with 7q. However, the three groups were otherwise similar in regard to other laboratory and clinical features, including overall survival (23). These findings are consistent with the results of large study involving a cohort of 1,593 MDS patients (944 Germans and 695 Japanese). In this study, clinical outcomes of der(1;7)(q10;p10) patients were significantly better than those having−7/del(7q) or 1q gain alone. Interestingly, der(1;7)(q10;p10) was found to be 10 times more frequent in Japanese than in Germans (4.5 vs. 0.43%) and the strong male predominance (86% of cases) of this entity was also confirmed (Okuda et al. The 80th annual meeting of the Japanese Society of Hematology, 2018, abstract OS3-5C-3).

Most MDS patients develop symptoms related to cytopenias and anemia, although isolated neutropenia and thrombocytopenia can also occur. In addition, some MDS patients may also present with eosinophilia (7). Among 288 patients with *de novo* MDS retrospectively analyzed by Matsushima and colleagues, 36 (12.5%) fulfilled the criterion for BM eosinophilia (eosinophils in BM exceeding 5%) and those with BM eosinophilia showed a higher tendency to evolve to AML and had a worst overall survival. In the same study, specific cytogenetic aberrations, especially, abnormalities in chromosome 7, complex karyotypes and i(17q), were associated with an increase in BM eosinophils (24). Nonetheless, it must be noted that the frequency of eosinophilia in MDS der(1;7)(q10;p10) has not been comprehensively investigated, likely due to the rarity of this entity. In the study by Slovak and colleagues, none of the 12 MDS patients with der(1;7)(q10;p10) showed eosinophilia (17). On the other hand, Sanada and colleagues documented eosinophilia in the peripheral blood of six out of 77 patients with der(1;7)(q10;p10), however none of those patients had eosinophilia in the BM (15).

The optimal treatment for MDS patients with der(1;7)(q10;p10) is another important aspect that has not been defined and patients are currently managed following the current treatment algorithm for MDS. In isolated case reports, the response with AZA was good (Imi et al. The 75th annual meeting of the Japanese Society of Hematology, 2013, abstract PS2-90), although the limited number of cases and the lack of controlled studies is a handicap to assume that AZA is the optimal treatment for this entity. In the cases reported here, AZA was ineffective in case 1 and by the time of diagnosis of case 2, AZA has not been approved for clinical use in Japan.

Corticosteroids are the first line therapy for all types of hypereosinophilia preventing end-organ damage caused by these disorders, however recent studies suggest that certain subtypes are less responsive to these agents and many either exhibit resistance or relapse during steroid tapering or withdrawal (4, 25, 26), as observed in the case presented here. On the other hand, tyrosine kinase inhibitors are highly effective in those cases harboring fusion gene as a cause of hypereosinophilia. For example, imatinib is the drug of choice for patients with FIP1L1-PDGFRA and ruxolitinib is the first drug of choice in hypereosinophilia driven by gain of function mutations involving JAK/STAT axis (4, 25, 26). None of those mutations were detectable in the cases presented here. Novel monoclonal antibodies capable of depleting circulating eosinophils, such as mepolizumab (targeting IL-5), omalizumab (anti-IgE), and dupilumab (anti-IL4α receptor subunit) have been recently approved by the FDA have therapeutic potential for the management of hypereosinophilia, especially those cases refractory to corticosteroids and not associated with tyrosine kinase mutations (25, 26).

Does der(1;7)(q10;p10) translocation play a role in the pathogenesis of MDS or in the development of eosinophilia? So far, no specific molecular targets have been identified for this translocation and it is unknown if the loss of 7q and/or gain of 1q play a direct role in the pathogenesis of this entity. It is plausible that those chromosomal aberrations may generate genetic rearrangement of genes encoding eosinophilopoietic cytokines. Alternatively, prominent eosinophilia has been reported in myeloid leukemia with translocations or deletions of chromosome 7 (27). Interestingly, the deletion of the long arm of chromosome 7 (7q) has been reported in association with eosinophilia in isolated cases of myeloid malignancies such as myelomonocytic leukemia with BM eosinophilia (28).

MDS with der(1;7)(q10;p10) has distinctive clinical and pathological characteristics, however, this translocation is found in a very small fraction of patients, and its pathogenic relevance to MDS and eosinophilia is unclear. Newer high-resolution whole-genome molecular approaches, such as comparative genomic hybridization and gene expression microarray studies, are expected to provide a more comprehensive analysis to unravel a potential role of this particular cytogenetic abnormality in disease pathogenesis.

## Ethics Statement

This study was carried out in accordance with the recommendations of Kindai University ethical committee. The protocol was approved by the Kindai University ethical committee. Written informed consent in accordance with the Declaration of Helsinki was obtained from the patient for analysis, publication of this report, and any accompanying images.

## Author Contributions

SR collected information and data and wrote the manuscript. JE conceived the study and wrote the manuscript. YM collected data. HT collected and analyzed data. IM analyzed data and supervised the study.

### Conflict of Interest Statement

The authors declare that the research was conducted in the absence of any commercial or financial relationships that could be construed as a potential conflict of interest.
